# Analysis of proliferating neuronal progenitors and immature neurons in the human hippocampus surgically removed from control and epileptic patients

**DOI:** 10.1038/s41598-019-54684-z

**Published:** 2019-12-03

**Authors:** Tatsunori Seki, Tomokatsu Hori, Hajime Miyata, Michiyo Maehara, Takashi Namba

**Affiliations:** 1 Department of Histology and Neuroanatomy, Tokyo Medical University, Tokyo, 160-8402 Japan; 2 Department of Anatomy, Juntendo University School of Medicine, Tokyo, 113-8421 Japan; 3 Department of Neurosurgery, Tokyo Neurological Center Hospital, Tokyo, 134-0088 Japan; 4 Department of Neurosurgery, Tokyo Women’s Medical University, Tokyo, 162-8666 Japan; 5 Department of Neuropathology, Research Institute for Brain and Blood Vessels, Akita Cerebrospinal and Cardiovascular Center, Akita, 010-0874 Japan; 6 Central Clinical Laboratory, Tokyo Women’s Medical University, Tokyo, 162-8666 Japan; 7 Max Planck Institute of Molecular Cell Biology and Genetics, Dresden, 01307 Germany

**Keywords:** Neuronal development, Adult neurogenesis

## Abstract

Adult neurogenesis in the mammalian hippocampus is a well-known phenomenon. However, it remains controversial as to what extent adult neurogenesis actually occurs in the adult human hippocampus, and how brain diseases, such as epilepsy, affect human adult neurogenesis. To address these questions, we analyzed immature neuronal marker-expressing (PSA-NCAM+) cells and proliferating neuronal progenitor (Ki67+/HuB+/DCX+) cells in the surgically removed hippocampus of epileptic patients. In control patients, a substantial number of PSA-NCAM+ cells were distributed densely below the granule cell layer. In epileptic patients with granule cell dispersion, the number of PSA-NCAM+ cells was reduced, and aberrant PSA-NCAM+ cells were found. However, the numbers of Ki67+/HuB+/DCX+ cells were very low in both control and epileptic patients. The large number of PSA-NCAM+ cells and few DCX+/HuB+/Ki-67+ cells observed in the controls suggest that immature-type neurons are not recently generated neurons, and that the level of hippocampal neuronal production in adult humans is low. These results also suggest that PSA-NCAM is a useful marker for analyzing the pathology of epilepsy, but different interpretations of the immunohistochemical results between humans and rodents are required.

## Introduction

Animal studies have demonstrated that hippocampal adult neurogenesis occurs in the subgranular zone (SGZ) of the dentate gyrus (DG)^1–5^, which is a narrow band below the dentate granule cell layer (GCL). The SGZ contains neural stem cells that give rise to proliferating early intermediate neuronal progenitors expressing neurogenin2, neuroD, and Hu, and these early intermediate progenitors then differentiate into proliferating late intermediate neuronal progenitor cells expressing immature neuronal markers, such as polysialylated neural cell adhesion molecule (PSA-NCAM)^2,3,6^ and doublecortin (DCX)^5,7,8^. After exiting the cell cycle, postmitotic immature neurons continue to express these immature neuronal markers for several weeks in rodents^9,10^ or several months^11,12^ in primates. Hippocampal adult neurogenesis is involved in various hippocampal functions, such as memory and learning^4,5,13,14^, and are affected by various physiological and pathological conditions^4,5,15,16^.

The existence of adult neurogenesis in humans was demonstrated in the late 1990’s^17^. Subsequently, many studies using immunohistochemistry and new techniques have been performed, but have provided conflicting results regarding the extent to which adult neurogenesis occurs^18,19^. Some immunohistochemical studies have suggested that the level of human hippocampal neurogenesis declines after birth and becomes low^20,21^ or undetectable^22^ in adults, whereas recent studies reported that hippocampal neurogenesis persists in adults^23–25^. Furthermore, studies using ^14^C incorporation suggest that persistent adult neurogenesis, comparable to that in middle-aged mice, occurs in adult humans^26,27^. Accumulating data suggest the involvement of adult human neurogenesis in some diseases^4,16^, such as epilepsy, ischemia, Parkinson disease, Alzheimer’s disease, depression^28^ and Huntington disease; however, there are controversial results regarding the effects of these diseases on adult neurogenesis.

Temporal lobe epilepsy (TLE) is the most common type of focal epilepsy, and is known to cause structural changes in the hippocampus of patients^29,30^. In the DG, dispersion of granule cells and loss of hilar neurons are well known pathological abnormalities^30,31^. In a rodent model of TLE, the level of adult neurogenesis in the DG is increased in acute seizure conditions^32–34^, but is reduced in severe or prolonged seizure conditions^34–36^, suggesting that alterations of adult neurogenesis are dependent on the severity and duration of the seizures. In addition, epileptic seizures have been shown to induce aberrant basal dendrites and ectopic migration to the hilus or molecular layer^34,37–39^. In epileptic patients, it has been reported that seizures enhance the level of neurogenesis in some cases, particularly in young patients^40^, but not in other cases^16,41,42^.

The discrepancies among these human studies appear to be owing to differences in specimens, fixation methods, and antibodies used to detect the neuronal progenitors and immature neurons^24,43^. In our present study on epileptic patients, we used hippocampal tissue specimens that had been surgically removed and then immediately fixed. To detect immature-type neurons, an antibody to PSA-NCAM was used. To detect proliferating intermediate progenitor cells, antibodies to HuB and DCX together with the cell proliferation marker Ki67 were used^8,44,45^, because a substantial number of Ki67+ neuronal progenitor cells express Hu and DCX^6,46^. Mammalian Hu proteins are a group of RNA-binding proteins that comprise four family members (HuR, HuB, HuC, and HuD). HuB, HuC, and HuD are expressed from early intermediate neuronal progenitor cells to mature neurons^45,46^ and regulate neuronal development^47^. Our present study demonstrates that a substantial number of PSA-NCAM-positive (PSA-NCAM+) cells exist in the human SGZ and hilus, and severe epilepsy causes structural changes in PSA-NCAM+ cells, but the number of DCX+/HuB+/Ki67+ proliferating intermediate progenitor cells is very low, suggesting that the majority of immature neuronal marker-positive neurons are not recently generated neurons, but show pathological alterations similar to those seen in newly generated neurons of rodents.

## Results

### PSA-NCAM expression in the adult human DG

To assess the severity of epilepsy, the cellular architecture in the DG of both control and epileptic patients was analyzed by Nissl staining. Furthermore, the expression pattern of the immature neuronal marker PSA-NCAM was analyzed in a section close to the Nissl-stained section from the same specimens.

**Figure 1:**
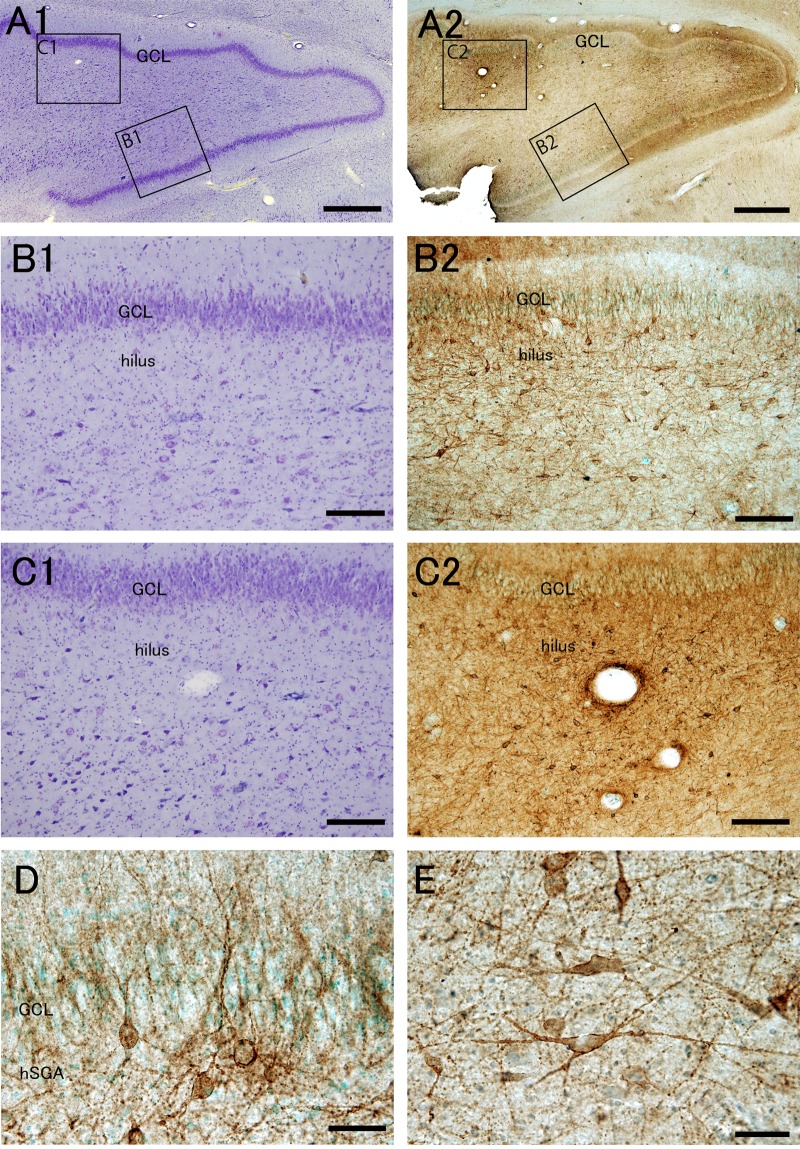
PSA-NCAM+ cells with various morphologies exist in the human subgranular area (hSGA) and hilus of control patients. Nissl staining (**A1–C1**) and PSA-NCAM immunohistochemistry with methyl green nuclear staining (**A2–C2,D,E**) in the dentate gyrus (**A–D**, control patient CN5; (**E)**, control patient CN6, see Supplemental Table 1). (**A–C**) The boxed regions in (**A1**,**A2**) are enlarged in (**B1**,**C1)**, and (**B2**,**C2)**, respectively. (**D**) PSA-NCAM expression in the granule cell layer (GCL) and hSGA. (**E**) PSA-NCAM expression in the hilus. Scale bars = 1 mm in (**A1**,**A2**); 200 μm in (**B1**,**B2**,**C1**,**C2**); and 50 μm in (**D**,**E)**.

In control patients, Nissl staining showed that granule cells were tightly packed in the GCL, and large neurons were distributed in the area enclosed by the C-shaped GCL, which consists of the hilus (also called polymorphic layer), CA4, and a part of the CA3 pyramidal cell layer (Fig. 1A1–C1, and Supplemental Fig. 1). PSA-NCAM immunohistochemistry demonstrated that a number of PSA-NCAM+ cells were densely present in a broad area below the GCL (Fig. 1A2,B2), and at times the area extended to the center region (Fig. 1A2,C2), which appeared to correspond to the hilus^48^. The distribution pattern of PSA-NCAM+ cells in the human DG was different from that of rodents (Supplemental Fig. 2). In rodents, PSA-NCAM+ cells are confined to the narrow SGZ, whereas in humans they were broadly distributed in the putative SGZ and hilus. Furthermore, the border between the putative SGZ and hilus was not clear in the human DG compared with that in rodents. Therefore, in the present study, the human SGZ was tentatively defined as a 50-μm-thick region below the GCL (Supplemental Fig. 2,B1,B2), because this area predominantly contained PSA-NCAM+ small or medium-sized round or spindle-shaped cells, which resembled rodent PSA-NCAM+ neural progenitor cells and immature neurons (Fig. 1B2,D, and Supplemental Fig. B2), and included much fewer large pyramidal cells and multipolar polygonal cells, as often observed in the deeper hilar region (Fig. 1B2,E, and Supplemental Fig. B2). For this reason, we refer to the putative human SGZ as the human subgranular area (hSGA), to distinguish it from the narrow and distinct SGZ of rodents. Additionally, ubiquitous PSA-NCAM immunoreactivity and scattered immunopositive cells were observed in the entire hippocampal formation.

In the hSGA, PSA-NCAM+ cells were mainly small or medium-sized round or spindle-shaped cells with tangentially or obliquely oriented apical dendrites (Fig. 1B2,D). Many apical dendrites arising from these cells transversed the GCL and reached the outer molecular layer. No PSA-NCAM+ thick mossy fiber bundles were found, unlike in rodents (Supplemental Fig. 2A2). Sometimes two adjacently located PSA-NCAM+ cells were found, but clusters of PSA-NCAM+ cells, as seen in the rodent SGZ were not detected, suggesting the possibility that the proliferative activity of PSA-NCAM+ cells is not high. A few pyramidal cells with apical and basal dendrites were also seen. In this respect, it was reported that normal human dentate granule cells have basal dendrites^49^. The hilus proper contained PSA-NCAM+ large or medium-sized pyramidal, polygonal, and horizontally oriented bipolar cells that extended straight processes (Fig. 1A2–C2,E). In addition, fewer number of PSA-NCAM+ cells were also observed in the CA4 and CA3.

**Figure 2:**
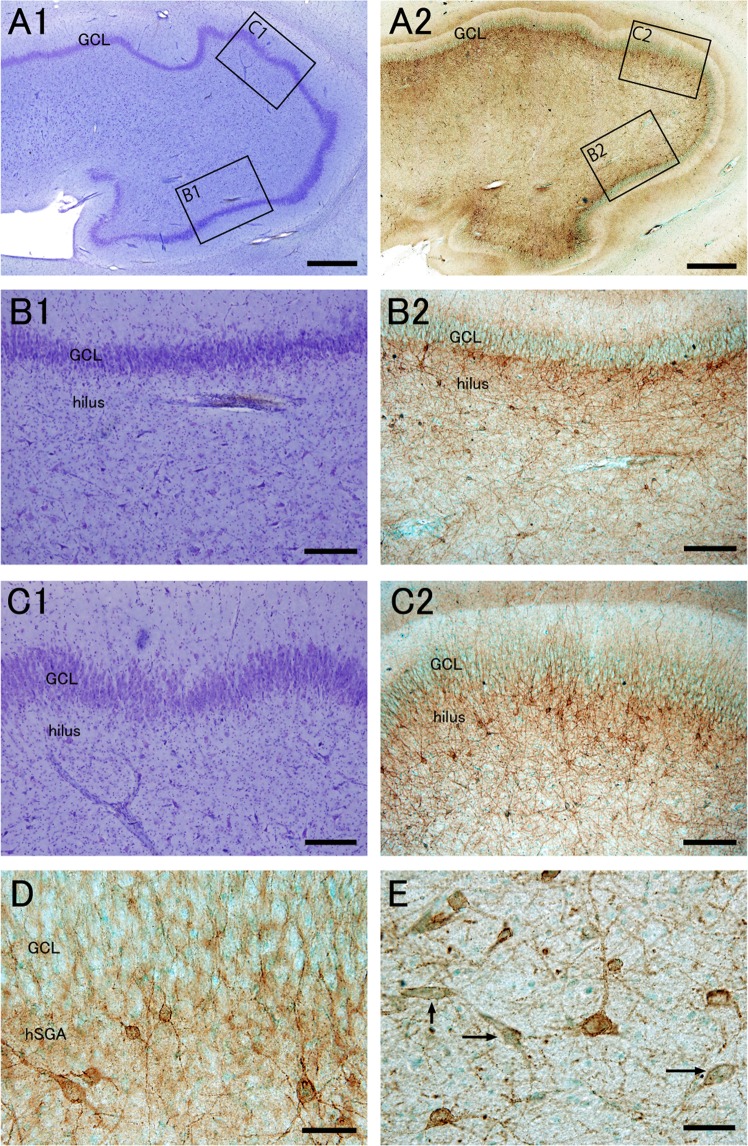
Distribution pattern and morphology of PSA-NCAM+ cells of epileptic patients without granule cell dispersion are similar to those of control patients. Nissl staining (**A1–C1**) and PSA-NCAM immunohistochemistry with methyl green nuclear staining (**A2–C2**,**D,E**) in the dentate gyrus of an epileptic patient (EP3, see Supplemental Table 1) without granule cell dispersion. (**A–C**) The boxed regions in (**A1**,**A2**) are enlarged in (**B1**,**C1**), and (**B2**,**C2**), respectively. (**D**) PSA-NCAM expression in the granule cell layer (GCL) and human subgranular area (hSGA). (**E**) PSA-NCAM+ cells in the hilus. Some PSA-NCAM+ cells have attenuated PSA-NCAM expression (arrows). Scale bars** = **1 mm in (**A1**,**A2**); 200 μm in (**B1**,**B2**,**C1**,**C2**); and 50 μm in (**D**,**E)**.

**Figure 3:**
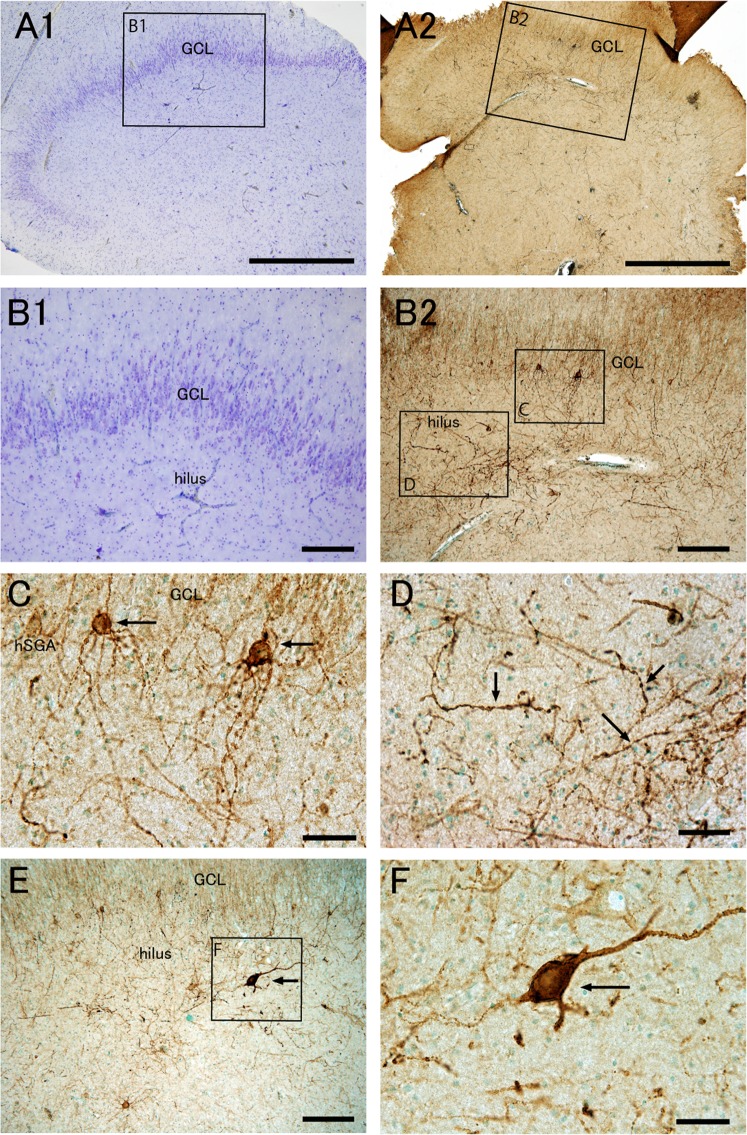
Epileptic patients with granule cell dispersion and loss of hilar neurons have aberrant PSA-NCAM+ cells with multibasal dendrites in the hSGA, and unusually large PSA-NCAM+ cells and thick fibers with varicosities in the hilus. Nissl staining (**A1**,**B1**) and PSA-NCAM immunohistochemistry with methyl green nuclear staining (**A2**,**B2**,**C–F**) in the dentate gyrus of an epileptic patient (EP9, see Supplemental Table 1) with granule cell dispersion and loss of hilar neurons. The boxed regions in (**A1**,**A2**,**B2**,**E)** are enlarged in (**B1**,**B2**,**C,D**,**F)**, respectively. Note the PSA-NCAM+ cells with multi-basal dendrites in the human subgranular area (hSGA) (**B2**,**C**), thick fibers with varicosities (**B2**,**D**), and strongly PSA-NCAM+ large cells (**E**,**F**) in the hilus. GCL, granule cell layer. Scale bars = 1 mm in **A1** and **A2**; 200 μm in (**B1,B2,E)**; and 50 μm in (**C,D,F)**.

In epileptic patients, Nissl staining demonstrated pathological changes in the DG. According to the structural features, epileptic patients were divided into the following two groups: one group without dispersion of granule cells (Fig. 2A1–C1), and the other group with dispersion and loss of large cells in the hilus (Figs. 3A1,B1, 4A1,B1).

**Figure 4:**
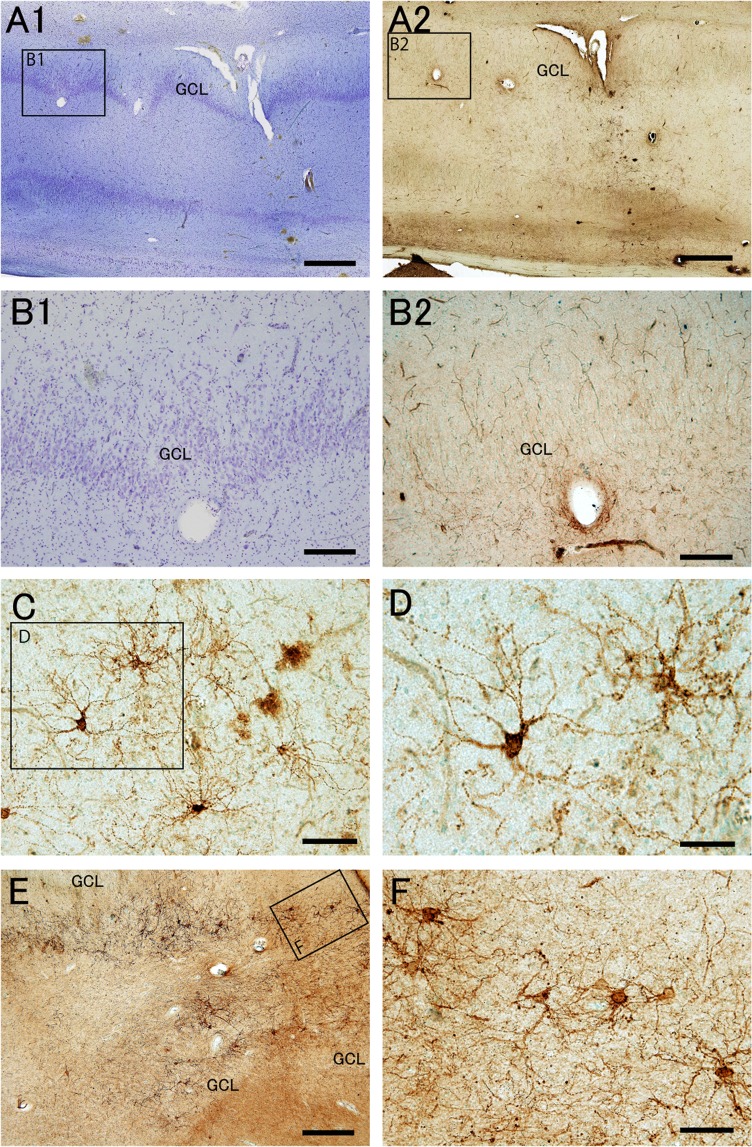
Epileptic patients with granule cell dispersion and loss of hilar neurons have aberrant PSA-NCAM+ cells with multipolar processes in the hilus. Nissl staining (**A1**,**B1**) and PSA-NCAM immunohistochemistry with methyl green nuclear staining (**A2**,**B2**,**C–F**) in the dentate gyrus of epileptic patients with granule cell dispersion and loss of hilar neurons (**A–D**, epileptic patient EP10; (**E**,**F)**, epileptic patient EP7, see Supplemental Table 1). The boxed regions in (**A1**,**A2**,**C,E)** are enlarged in (**B1**,**B2**,**D,F)**, respectively. Note the reduction in the number of PSA-NCAM+ cells in the human subgranular area (hSGA) and hilus (**B2**) and the presence of aberrant PSA-NCAM+ cells with multipolar processes (**C–F**). GCL, granule cell layer. Scale bars = 1 mm in (**A1**,**A2**); 500 μm in (**E)**; 200 μm in (**B1**,**B2**); 100 μm in (**C**,**F)**; and 50 μm in **(D)**.

In patients without dispersion of granule cells, the distribution pattern and morphology of PSA-NCAM+ cells was largely similar to that of control patients (Fig. 2A2–C2,D,E). PSA-NCAM+ cells mainly composed of round and spindle-shaped cells were observed in the hSGA (Fig. 2B2,C2,D), and pyramidal, polygonal and bipolar cells with straight processes were present in the hilus (Fig. 2B2,C2,E).

In patients with dispersion of the GCL, loss of hilar neurons, and hippocampal sclerosis (Fig. 3A1,B1, 4A1,B1, and Supplementary Table 1), the numbers of PSA-NCAM+ cells in both the hSGA and hilus appeared to be decreased (Figs. 3A2,B2, 4A2,B2). In the hSGA, a few moderately or weakly PSA-NCAM+ small round or spindle-shaped cells sometimes remained just below the GCL (Fig. 3A2,B2,E). However, the most prominent feature was several types of abnormal cells expressing PSA-NCAM; round cells with many thin and long beaded basal dendrites in the hSGA (27% of total PSA-NCAM+cells in the hSGA; Fig. 3A2–C2), spindle or pyramidal-shaped cells with thick straight or tortuous processes (24% of total PSA-NCAM+ cells in the GCL-enclosed area; Fig. 3E,F), and multipolar cells with many radially oriented thin beaded processes with many ramifications (23% of total PSA-NCAM+ cells in the GCL-enclosed area; Fig. 4C–F). In the hilar region, many thick tortuous processes were found in all patients with dispersion of the GCL, loss of hilar neurons, and hippocampal sclerosis (Fig. 3B2,D). Further analysis of these aberrant PSA-NCAM+ cells demonstrated that they were negative for glial fibrillary acidic protein (GFAP), and had glutamic acid decarboxylase 65-positive terminals on the soma and processes, suggesting that aberrant PSA-NCAM+ neurons are integrated into hippocampal neuronal circuits (Supplemental Fig. 3).

**Figure 5:**
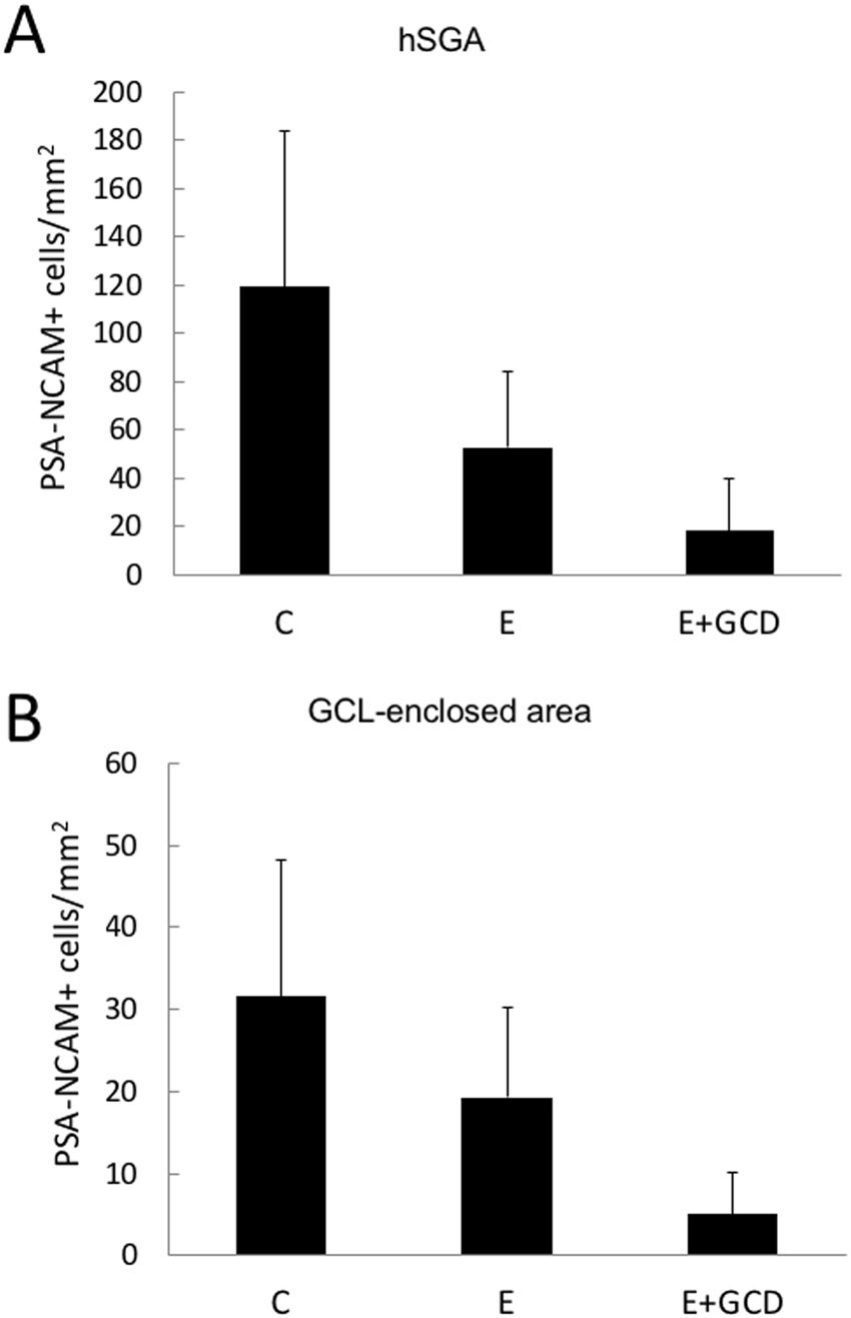
Numbers of PSA-NCAM+ cells in the GCL-enclosed area and the hSGA are significantly decreased in E+ GCD patients compared with control patients. Quantitative analysis of PSA-NCAM-positive (PSA-NCAM+) cells in the dentate gyrus of control patients (C, n = 6), epileptic patients without granule cell dispersion (E, n = 6) and epileptic patients with granule cell dispersion (E + GCD, n = 5). The number of PSA-NCAM+ cells were counted in the human subgranular area (hSGA, **A**) and the granule cell layer (GCL)-enclosed area (**B**). (*P < 0.01, one-way ANOVA followed by the Tukey–Kramer test).

Next, the number of PSA-NCAM+ cells was quantitatively compared among control patients and epileptic patients with or without granule cell dispersion (Fig. 5). PSA-NCAM+ cells were counted in the hSGA and an area enclosed by the C-shaped GCL consisting of the hilus, CA4 and a part of the CA3 pyramidal cell layer (GCL-enclosed area), because unlike the rodent hippocampus, in humans, PSA-NCAM+ cells are distributed in the entire GCL-enclosed area, and it is sometimes difficult to differentiate cells of the hilus from CA4 cells (Supplemental Fig. 1). In patients without dispersion of granule cells, the numbers of PSA-NCAM+ cells in both the hSGA (n = 6, 53 ± 32 cells/mm^2^) and the GCL-enclosed area (n = 6, 19 ± 11 cells/mm^2^) were decreased by 39% and 56%, respectively, when compared with control patients (n = 6; hSGA, 119 ± 65 cells/mm^2^; GCL-enclosed area, 31 ± 17 cells/mm^2^); however, there was no statistically significant difference between controls and patients without dispersion of granule cells (Fig. 5). Significant differences in the number of PSA-NCAM+ cells in the hSGA and the GCL-enclosed area were detected between controls (n = 6; hSGA, 119 ± 65 cells/mm^2^; GCL-enclosed area, 31 ± 17 cells/mm^2^) and patients with dispersion of granule cells and loss of hilar neurons (n = 5; hSGA, 18 ± 21 cells/mm^2^, *P* < 0.01; GCL-enclosed area, 5 ± 5 cells/mm^2^, *P* < 0.01).

Because PSA-NCAM+ cells in epileptic patients appeared to be larger than those in control subjects, we measured the size of the PSA-NCAM+ cells. PSA-NCAM+ cells were larger in epileptic patients with granule cell dispersion (n = 5, 293 ± 149.2 μm^2^) than controls (n = 6, 217.2 ± 61.6 μm^2^, control vs E + sGCD, *P* < 0.01), and epileptic patients without granule cell dispersion (n = 6, 238.3 ± 92.9 μm^2^, E vs E + sGCD, *P* < 0.01), suggesting that severe epilepsy causes the swelling of PSA-NCAM+ cells. Additionally, we performed correlation analysis between the age and number of PSA-NCAM+ cells in the hSGA and GCL-enclosed area of control and epileptic patients, but did not find any significant correlation.

### Proliferating neuronal progenitor cells in the adult DG

Before performing quantitative analysis of proliferating neuronal progenitors, some preliminary experiments were performed to analyze the optimal conditions of immunohistochemistry. For Ki67 immunohistochemistry, pretreatment with citrate buffer (pH 6.0) at 98 °C or 121 °C was necessary, but these treatments abolished PSA-NCAM immunoreactivity (Supplemental Fig. 4A)^50^. For this reason, PSA-NCAM immunohistochemistry could not be performed together with Ki67 immunohistochemistry. Instead, an antibody against DCX, a well-known marker for late proliferating neuronal progenitor cells and immature neurons was used.

In the next preliminary experiments, the distribution patterns of PSA-NCAM+ cells and DCX+ cells were compared by double immunohistochemistry for PSA-NCAM and DCX without citrate buffer pretreatment. The distribution of PSA-NCAM+ cells and DCX+ cells appeared to largely overlap (Supplemental Fig. 4B), but it was sometimes difficult to distinguish DCX+ cells from non-specific staining, because DCX immunoreactivity was seen as aggregates only in the soma, but not in the dendrites (Supplemental Fig. 4C). A similar immunostaining pattern has been reported in studies without antigen retrieval procedures in the adult human GCL^20,21,51^.

**Figure 6:**
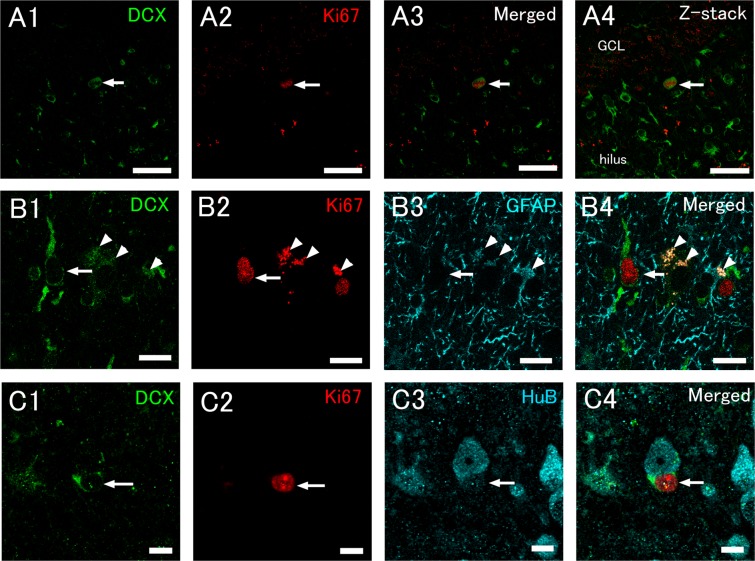
DCX and Ki67 immunohistochemistry with antigen retrieval can detect cell bodies and processes of proliferating neuronal precursors, but may also recognize non-neuronal cells, such as satellite cells. A DCX+ cell expressing Ki67 in the human subgranular area (hSGA) (arrows, **A**). Note that many cells show clear cytoplasmic DCX expression. DCX and Ki67 double-positive cells are devoid of GFAP expression (arrows, **B**). Arrowheads in (**B)** indicate lipofuscin granules. Z-stack images of A4 were reconstructed from 11 optical slices, respectively. Images A and B were obtained from control patient CN2 (see Supplemental Table 1). (**C**) A Ki67+/DCX+ satellite cell (arrows) in the hSGA of an epileptic patient (epileptic patient EP8). Scale bars = 50 μm in (**A**); 20 μm in (**B**); and 10 μm in (**C**).

On the other hand, in the case of DCX immunohistochemistry performed with antigen retrieval using citrate buffer or Liberate Antibody Binding (L.A.B.) solution, weak or moderate DCX immunoreactivity was uniformly distributed in both the cytoplasm of the soma and proximal processes (Fig. 6A,B), suggesting that DCX immunostaining with antigen retrieval pretreatment is a reliable method to detect immature neuronal marker-expressing cells in human hippocampi under the present experimental condition.

Furthermore, triple immunohistochemistry for Ki67, DCX, and GFAP showed that as far as we observed, most DCX+/Ki67+ cells did not show GFAP expression (Fig. 6B), suggesting that proliferating DCX+ cells are not astrocytes. However, a few small DCX+/Ki67+ cells without HuB expression were found to exist adjacently to large HuB+ granule cells, and appeared to be satellite cells judging from their size, shape, and location^52^ (Fig. 6C). Because satellite cells are generally considered to be glial cells^52^, the possibility of the presence of DCX+ satellite glial cells cannot be excluded. For this reason, Ki67+/DCX+ cells were not counted as neuronal progenitor cells in our present study.

**Figure 7:**
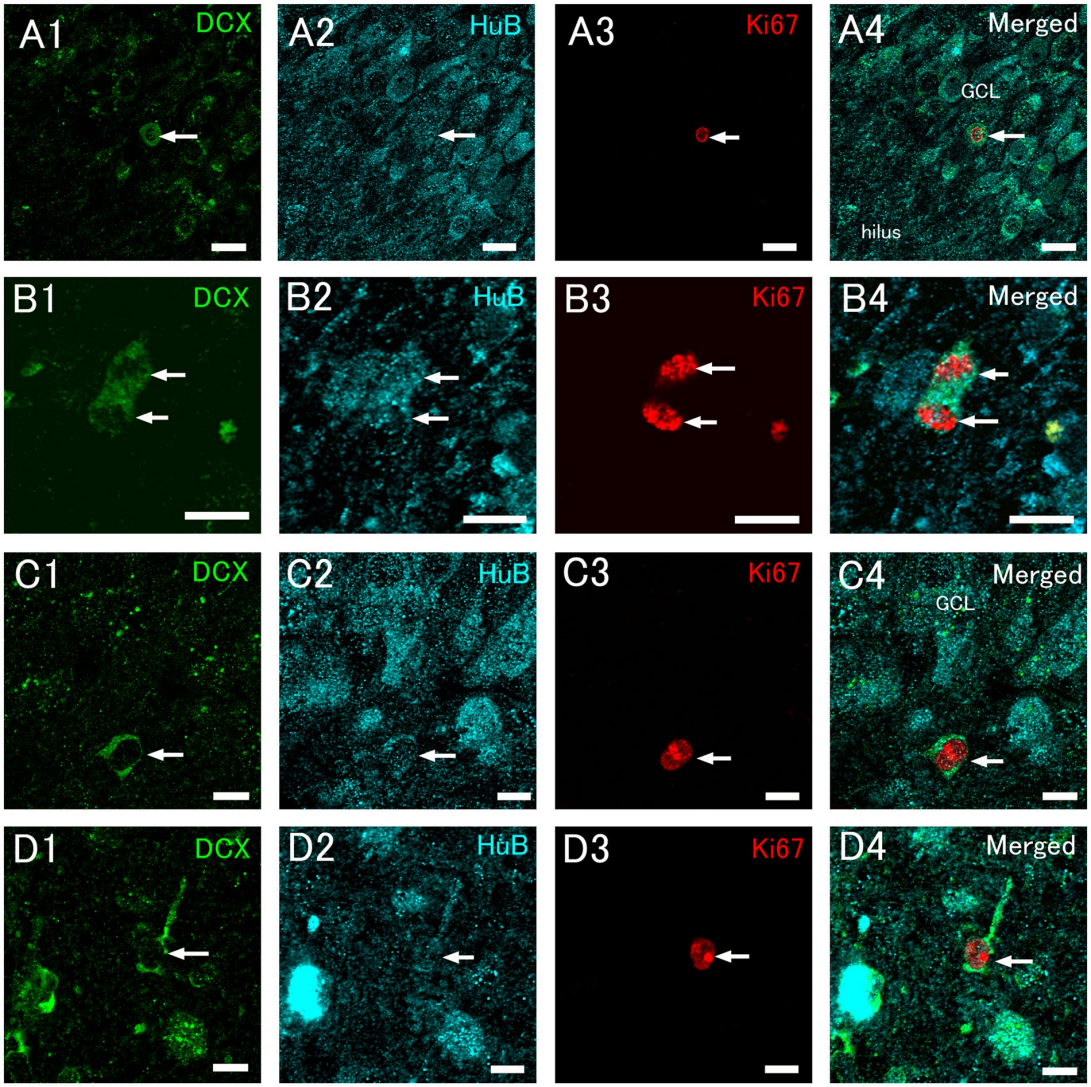
Triple immunohistochemical staining for DCX, HuB, and Ki67 detects proliferating neuronal progenitor cells. (**A,B**) Triple-positive cells (arrows) in the human subgranular area (hSGA, **A**) and hilus (**B**) of a control patient (CN2, see Supplemental Table 1). (**C,D**) Triple-positive cells (arrows) in the hSGA (**C**) of an epileptic patient (EP5), and in the hilus (**D**) of an epileptic patient (EP8). (**E**) A Ki67+/HuB+ cell (arrows) in the hSGA of an epileptic patient (EP8). Scale bars = 20 μm in (**A**); and 10 μm in (**B–E**).

In the next quantitative analysis, in addition to the anti-DCX antibody, the anti-HuB antibody was used for detecting neuronal progenitor cells and immature neurons^44,46^. This is because HuB has been found to be a reliable marker for neuronal progenitors in rodent adult neurogenesis^45,46^, and is not expressed by satellite glial cells (Fig. 6C). Ki67+/HuB+/DCX+ triple-positive cells were found in the hSGA and the hilus of both control and epileptic patients (Fig. 7). These were round or ovoid cells that sometimes had a short process (Fig. 7D). The intensity of HuB and DCX immunoreactivity was generally weak or moderate. These triple-positive cells, which sometimes had condensed chromatin (Fig. 7B) or were closely apposed to each other (Fig. 7B,C), were found in the hSGA (Fig. 7C) and hilus (Fig. 7B), suggesting that they are dividing cells. The numbers of Ki67+/HuB+/DCX cells were very small (Table 1) and the presence of Ki67+/HuB+/DCX+ cells varied from patient to patient. We analyzed a total of 83 sections; control patients (total: 41 sections; average: 6.83 sections/patient), epileptic patients without granule cell dispersion (total: 21 sections; average: 3.5 sections/patient), and epileptic patients with granule cell dispersion (total: 22 sections, average: 4.4 sections/patient). Ki67+/HuB+/DCX+ cells were detected in 8 out of 17 subjects; 4 out of 6 subjects from the control group, 2 out of 6 subjects from the epileptic patients without cell dispersion of the GC, and 2 out of 5 subjects from epileptic patients with cell dispersion. The numbers of Ki67+/HuB+ cells were not significantly different among the controls, epileptic patients without dispersion of the GCL, and epileptic patients with cell dispersion (Table 1). The results of the proliferating neuronal progenitors suggest that neurogenic activity in the human DG is much lower than in young and middle-aged rodents, and this implies that PSA-NCAM+ cells and DCX+ cells are not neurons that have been generated recently.

**Table 1:** Number of HuB+/DCX+/Ki67+ cells in the hSGA and GCL-enclosed area.

	N	hSGA	GCL-enclosed area^d^
		Mean ± S.D^a^	Mean ± S.D^a^
Control	6	0.146 ± 0.260	0.097 ± 0.130
E^b^	6	0.150 ± 0.235	0 ± 0
E + GCD^c)^	5	0 ± 0	0.106 ± 0.152

^a^Number of triple-positive cells/section.

^b^Epileptic patients without granule cell dispersion.

^c^Epileptic patients with granule cell dispersion.

^d^Excluding the hSGA.

## Discussion

In the present study, dense populations of PSA-NCAM+ cells were observed in the hSGA, which is thought to correspond to the rodent SGZ, and the hilus of control patients, whereas a reduced number and aberrant morphology of PSA-NCAM+ cells were found in epileptic patients with dispersion of granule cells. Further analysis of proliferating neuronal progenitor cells using antibodies for Ki67, HuB, and DCX showed that the level of neuronal production is very low. These results suggest that PSA-NCAM+ is a useful marker for neuropathological analyses of epileptic patients, but unlike rodents, most PSA-NCAM+ cells are not recently generated cells in the human hippocampus. In this discussion we will initially discuss the discrepancy between the existence of a substantial number of PSA-NCAM+cells and very low neurogenic activity in the adult human hippocampus, and alterations of these cells in epileptic patients.

The morphology and distribution pattern of PSA-NCAM+ cells in the adult DG has been repeatedly reported in rodents^2–6^. However, it has remained unclear as to whether they share similarities with PSA-NCAM+ cells in the human DG. In the adult rodent DG, PSA-NCAM+ cells, which are newly generated granule cells, are principally confined to the narrow SGZ and innermost part of the GCL, and comprise mainly small or medium-sized round or ovoid-shaped cells with radially oriented long apical dendrites, partially, horizontally oriented spindle-shaped cells with long horizontal processes, and small round cells with short processes^2,6,53^. Additionally, proliferating and postmitotic PSA-NCAM+ cells often form clusters together with proliferating early progenitor cells in the SGZ^6^.

Our present observations in the adult human DG demonstrated that PSA-NCAM+ cells are distributed in a wider area below the GCL comprising the hSGA and hilus. The hSGA mainly contained cells similar to those observed in rodents, suggesting the possibility that they share common properties, such as plasticity and immaturity. However, unlike rodents, clusters of PSA-NCAM+ cells were rarely observed in humans, indicating the low proliferating activity of PSA-NCAM+ cells. The human hilus included different types of PSA-NCAM+ cells, such as large pyramidal cells and multipolar cells that did not appear to be newly generated cells or immature cells. In this respect, some previous studies have also reported the presence of a substantial number of PSA-NCAM+ cells in the human hilus^54,55^ in addition to the SGZ^22,23,54,55^. However, the properties of these hilar PSA-NCAM+ cells in humans have not been clarified.

Our analysis using Ki67, HuB, and DCX antibodies showed that unlike rodents, the number of proliferative neuronal progenitor cells was very low in the human DG. The low level of proliferating neuronal progenitor cells suggests that most of the observed PSA-NCAM+ cells are not cells that were recently generated in the human hippocampus, unlike in the rodent hippocampus. In this regard, there have been conflicting reports as to the extent to which neurogenesis occurs in the adult human hippocampus. Some immunohistochemical studies of postmortem brains have indicated that after birth, the numbers of DCX+cells and DCX+/Ki67+ cells in the DG decline exponentially, and become very low by 2 to 3 years of age^20,21^. Therefore, it is likely that the number of Nestin+ progenitor cells in the human GCL are decreased during fetal maturation to undetectable levels within one year after birth^40^. A recent study in the postmortem brain and surgically resected samples from epileptic patients showed that the number of Ki67+/Sox2+ proliferating progenitors and DCX+/PSA-NCAM+ young neurons in the DG decline sharply during the first year of life, and that young neurons are not detected in adults^22^.

Other reports have demonstrated that persistent neurogenesis occurs in the adult human hippocampus. A recent immunohistochemical study on hippocampi collected on autopsy demonstrate the persistence of proliferating neuronal progenitors and immature neurons, despite a decline in the number of quiescent stem cells^23^. Another recent immunohistochemical study using improved tissue processing methods showed that adult neurogenesis occurred frequently^24^. Furthermore, new approaches by measuring the concentration of nuclear-bomb-test-derived ^14^C in genomic DNA demonstrated that a substantial number of new neurons are added to the human hippocampus every day, suggesting that humans and mice have similar levels of adult neurogenesis^26^.

The discrepancy in immunohistochemical studies appears to be caused by differences in specimens and techniques^24,43^. In the above-mentioned previous studies, the specimens were derived from postmortem brains with different postmortem intervals until fixation or different methods of surgical removal of tissue. Regarding immunohistochemistry, there are differences in the methods of tissue preparation among the samples (paraffin or cryostat sections), and immunohistochemical procedures varied among the samples (with/without antigen retrieval pretreatment). In our present study, the specimens surgically removed and immediately fixed were fresh tissue, which may differ from specimens with long postmortem intervals. Furthermore, we have performed some preliminary experiments indicating the requirement of antigen retrieval treatments to clearly show DCX+ cells and their processes. Therefore, these are important issues to take into account^24,43,56,57^, when the conclusions on adult human neurogenesis in the previous and present studies are considered.

To assess the extent of adult human neurogenesis, data from non-human primates, in which bromodeoxyuridine (BrdU) analysis can be performed is important. Detailed quantitative studies using the BrdU-labeling technique have repeatedly indicated the age-associated exponential decline of adult non-human primate neurogenesis in common marmosets^58^ and macaque monkeys^12,59,60^. A comparison of the age-dependent decline in neurogenesis between rodents and non-human primates has resulted in an important hypothetical concept that the decline of neurogenesis, particularly proliferation of progenitor cells, is regulated by absolute age, and not by relative age^60,61^. If so, the level of adult human neurogenesis may be as low as in non-human primates, which is in agreement with our present conclusion.

Our present data suggest that most PSA-NCAM+ cells are not recently generated neurons. Then, what is the identity of the PSA-NCAM+ cells? There appears to be at least two possibilities. One is that the newly generated cells in the human DG have prolonged PSA-NCAM expression. In rodents, most newly generated neurons lose PSA-NCAM and DCX immunoreactivity by 1 to 1.5 months after cell division, indicating that the maturation of newly born granule cells occurs by 1 to 1.5 months^9,10^. However, in adult macaque monkeys, maturation of new granule cells at the structural and molecular levels takes more than 6 months^11,12^. This suggests that newly generated neurons in non-human primates possess high plasticity over a long period. This prolonged immature state may compensate for the decline in plasticity by a rapid decrease in neurogenesis in primates including humans, in terms of plasticity. Another possibility is that PSA-NCAM+ cells are not newly generated neurons, but retain some properties similar to immature neurons. In rodents, PSA-NCAM and DCX are also expressed by neurons that were not recently generated in the non-neurogenic region of the adult brain, such as the piriform cortex^62,63^, cingulate cortex^64,65^, brain stem^66^, and spinal cord^67^. These PSA-NCAM-expressing and DCX-expressing cells are thought to be in a state of arrested development^64^. Taken together, most immature neuronal marker-expressing cells in the human DG appear to be neurons that were not recently generated, but those that were born a long time previously and continue to express immature neuronal markers or re-express them at a postnatal time point. The nature and function of immature marker-expressing cells in the adult human DG remain to be solved.

In rodent models of chronic epilepsy, a decrease in the numbers of PSA-NCAM+ and DCX+ newly generated granule cells, and progenitor cells in the SGZ has been reported^34–36^, whereas in acute epilepsy models an increase in these cells has been reported^32–34,68,69^. The situation of epileptic patients appears to correspond to that of rodents with prolonged seizures, because the hippocampus is generally removed from epileptic patients after many years from epileptic seizure onset. In fact, a previous study^55^ and our present study have shown that in epileptic patients, the number of PSA-NCAM+ cells are reduced. Our present study suggests that a decrease in the number of PSA-NCAM+ cells is associated with the severity of epilepsy, such as granule cell dispersion and sclerosis. However, it remains unclear as to how acute epilepsy affects human adult neurogenesis. Additionally, aberrant basal dendrites were observed in the cells of the SGZ of epileptic patients with granule cell dispersion. In rodent models of epilepsy, similar basal dendrites are found in newly generated cells^34,37,38^. Taken together, although most PSA-NCAM+ cells in the hSGZ are unlikely to be recently generated cells, similar pathological alterations occur in PSA-NCAM+ cells in both rodents and humans, suggesting that human and rodent PSA-NCAM+ cells share similar properties regarding vulnerability to epileptic seizures and structural plasticity.

In conclusion, the present study of the human hippocampus suggests that adult neurogenesis occurs at a low level. This conclusion is supported by several previous studies in humans^20–22^ and non-human primates^12,60^, although the extent of neurogenesis occurring in the adult human hippocampus remains controversial^18,22–25,28,43^. The present results also indicate that most PSA-NCAM+ cells are not recently generated neurons. Nevertheless we found a similarity in the morphology, distribution, and epilepsy-induced alterations between human PSA-NCAM+ cells and rodent newly generated granule cells, suggesting that they may share some similar properties, in terms of immaturity. A substantial number of PSA-NCAM+ cells in the adult human hippocampus may compensate for the decline in plasticity by a low level of neurogenesis. The exact nature, functions, developmental state, and pathology of PSA-NCAM+ cells in the hSGA and hilus should be addressed in future research.

## Methods

### Tissue collection

A total of 18 hippocampi, which were surgically removed between 2004 to 2007 from patients in the Department of Neurosurgery,Tokyo Women’s Medical University, were analyzed. Prior to detailed analyses, the structure of 20 hippocampi and their immunoreactivity to the antibodies used for the analyses were investigated, and 18 specimens suitable for further analysis were selected, including 12 specimens from patients with TLE treated by selective amygdalohippocampectomy with or without temporal lobectomy, and 6 control specimens from patients in whom temporal lobes, including medial temporal structures, were surgically resected for the complete removal of gliomas and cavernous angiomas. For the control specimens, hippocampi without apparent glioma invasion were used. However, as the presence of a few glioma cells cannot be completely denied, the counting of Ki67 single-positive cells was not performed. The ratio of men to women was 14 to 3, average age at operation was 33 ± 11 years (9–49 years), and the side of the operation was the left in 9 patients, and the right in 8 patients. Operations on all patients were performed by a single neurosurgeon (T.H.) who belonged to Tokyo Women’s Medical University at the time. Immunostaining and histological analyses were performed at Juntendo University, to which two of the authors (T.S. and T.N.) belonged to at the time. This study was approved by the Medical Research Ethics Committees of Tokyo Women’s Medical University (study approval no. 625) and Juntendo University (study approval no. 251), and the methods were carried out in accordance with the Ethics Guidelines for Clinical Studies of the Ministry of Health, Labour and Welfare of Japan. Informed consent was obtained from participants or parents of participants under the age of 18 years. Demographic data of the patients are shown in Supplemental Table 1.

### Tissue preparations

Surgically removed tissue blocks containing the hippocampi of control and epileptic patients were immediately fixed within 5 minutes of removal in 4% paraformaldehyde in 0.1 M phosphate buffer (PB, pH 7.4) and kept in the same fixative in a shaker for 3 days at 4 °C. The hippocampus blocks were taken from the anterior to the middle part of the hippocampus. The blocks were washed with phosphate-buffered saline (PBS), placed in 10% and then 20% sucrose in PBS, embedded in OCT compound and stored at −80 °C until sectioning. The specimens were cut into 30-μm sections with a cryostat. The sections were kept until use in cryoprotectant solution (30% ethylene glycol, 25% glycerin, 45% 0.1 M PB) at −20 °C.

### Immunohistochemistry

Primary and secondary antibodies were diluted with 1% Triton X-100 in PBS (PBS-T) containing 1% bovine serum albumin. Each step was followed by washing with PBS-T.

For PSA-NCAM immunostaining, the sections were pretreated with 100% methanol containing 0.3% H_2_O_2_ for 30 minutes to improve penetration of the antibody into the tissue sections. The sections were first reacted with a mouse IgM monoclonal antibody to PSA-NCAM (12E3, 1:1,000)^62,70^ at 4 °C for 48 hours, and then incubated with biotinylated goat anti-mouse IgM (1:220, Vectastain) at room temperature for 1 to 2 hours. Next, the sections were incubated with avidin-biotin peroxidase complex (Vectastain ABC reagent). Sections were pre-incubated with 0.02% 3,3′-diaminobenzidine tetrahydrochloride (DAB) in 0.05 M Tris buffer (pH 7.6) for 30 minutes and then in the DAB solution with 0.005% H_2_O_2_ for 5 to 10 minutes. The sections were counterstained with methyl green.

For immunostaining for Ki67, DCX, and Hu, sections were treated either with 0.01 M citrate buffer (pH 6.0) at 98 °C for 10 minutes in a thermostat bath, 0.01 M citrate buffer (pH 6.0) at 121 °C for 10 minutes in an autoclave or in L.A.B. solution (Polysciences) at 60 °C for 15 minutes in a thermostat bath to expose primary antigen binding sites. Sections were incubated with a mixture of primary antibodies at 4 °C for 1 to 2 overnights and then with a mixture of secondary antibodies at room temperature for 1 to 2 hours. The slides were covered with a solution of 30% ethylene glycol, 25% glycerin, and 45% 0.1 M PB containing 4’,6-diamidino-2-phenylindole.

Sections were examined using an Olympus BX53 microscope with a DP80 CCD camera, or a Zeiss confocal laser-scanning microscope 510 META (Carl Zeiss, Oberkochen, Germany), and in some cases, fluorescence images were digitally zoomed at 0.5× to 2×, and stacks of optical sections (0.9 to 1.8 μm in thickness) were obtained at 0.45 and 0.9 μm increments on the z-axis, respectively. Tiling images were produced with ZEN processing software (Zeiss). The images were corrected for brightness and contrast, and composed using the Zeiss LSM Image Browser and ZEN software, and Adobe Photoshop CS5 (Mountain View, CA). Images were processed and arranged at Juntendo University and Tokyo Medical University.

### Quantification and statistical analysis

In Fig. 5 and Table 1, all positive cells in a GCL-enclosed area of a section (2 to 5 sections for each individual) were counted, and the number of positive cells/section or area was used for statistical analysis. To measure the area of each PSA-NCAM+ cell in control and epileptic patients without seizures, three representative square images (1 mm^2^) in the GCL-enclosed area of each section were randomly collected. In the case of epileptic patients with severe seizures in which the number of PSA-NCAM+ cells was small, the cell area of all PSA-NCAM+ cells in the GCL-enclosed area of each section was measured. To measure an area, Image J software (NIH) was used. Data are shown as the mean ± SD; Statistical significance was assessed by one-way ANOVA followed by the Tukey–Kramer test (Fig. 4) or Kruskal-Wallis test (Table 1).

## Supplementary information

Supplementary Data
