# Supplementary material for: Analysis of proliferating neuronal progenitors and immature neurons in the human hippocampus surgically removed from control and epileptic patients
Source: Sci Rep. 2019 Dec 3;9:18194. doi: 10.1038/s41598-019-54684-z (PMC6890740; doi:10.1038/s41598-019-54684-z)
Supplement: Supplementary file 1 — Supplementary Data [file 41598_2019_54684_MOESM1_ESM.pdf]

Analysis of proliferating neuronal progenitors and immature neurons in the human hippocampus surgically removed from control and epileptic patients

Tatsunori Seki[1,2]*, Tomokatsu Hori[3,4], Hajime Miyata[5], Michiyo Maehara[4,6], and Takashi Namba[2,7]

[1]Department of Histology and Neuroanatomy, Tokyo Medical University, Tokyo 160-8402, Japan

[2]Department of Anatomy, Juntendo University School of Medicine, 113-8421 Tokyo Japan

[3]Department of Neurosurgery, Tokyo Neurological Center Hospital, Tokyo 134-0088, Japan

[4]Department of Neurosurgery, Tokyo Women's Medical University, Tokyo 162-8666, Japan

[5]Department of Neuropathology, Research Institute for Brain and Blood Vessels, Akita Cerebrospinal and Cardiovascular Center, Akita 010-0874, Japan

[6]Central Clinical Laboratory, Tokyo Women's Medical University, Tokyo 162-8666, Japan

[7]Max Planck Institute of Molecular Cell Biology and Genetics, Dresden 01307, Germany

***Corresponding author:**

Tatsunori Seki, Ph.D.
Department of Histology and Neuroanatomy
Tokyo Medical University
6-1-1 Shinjuku, Shinjuku-ku, Tokyo 160-8402, Japan
Phone: +81-(0)3-3351-6141 (ext. 274)
Fax: +81-(0)3-3351-7886
Email: sekit@tokyo-med.ac.jp

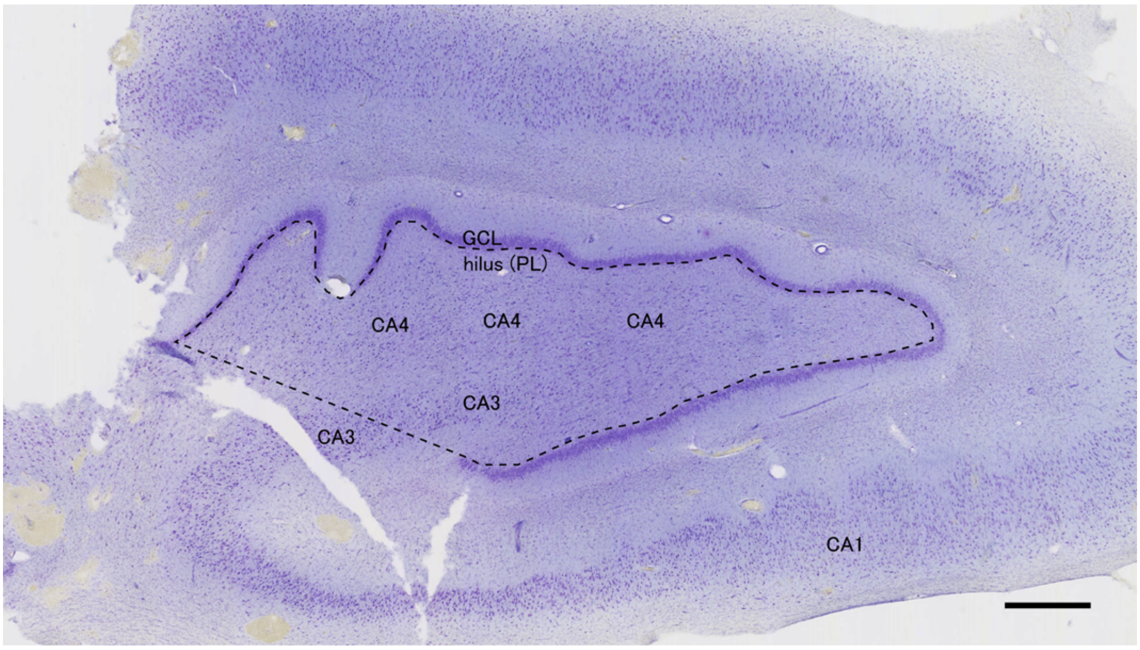

Supplemental Figure 1. Nissl staining of the hippocampal formation. The proximal tip of the CA3 pyramidal cell layer bends and forms the so-called end blade or CA4, and similar-looking large neurons are observed in both the CA4 and the CA3 regions. The hilus (also called polymorphic layer, PL) is located between the granule cell layer (GCL) and CA4, and appears to contain fewer number of large neurons, compared with CA4. In Figure 4, PSA-NCAM+ cells were counted in the hSGA (see Supplemental Figure 2) and an area enclosed by the C-shaped GCL consisting of the hilus and a part of the CA3 pyramidal cell layer (GCL-enclosed area, demarcated by a dashed line), because unlike the rodent hippocampus, in humans, PSA-NCAM+ cells are distributed in the entire GCL-enclosed area, and it is sometimes difficult to differentiate cells of the hilus from CA4 cells. Scale bar = 1 mm

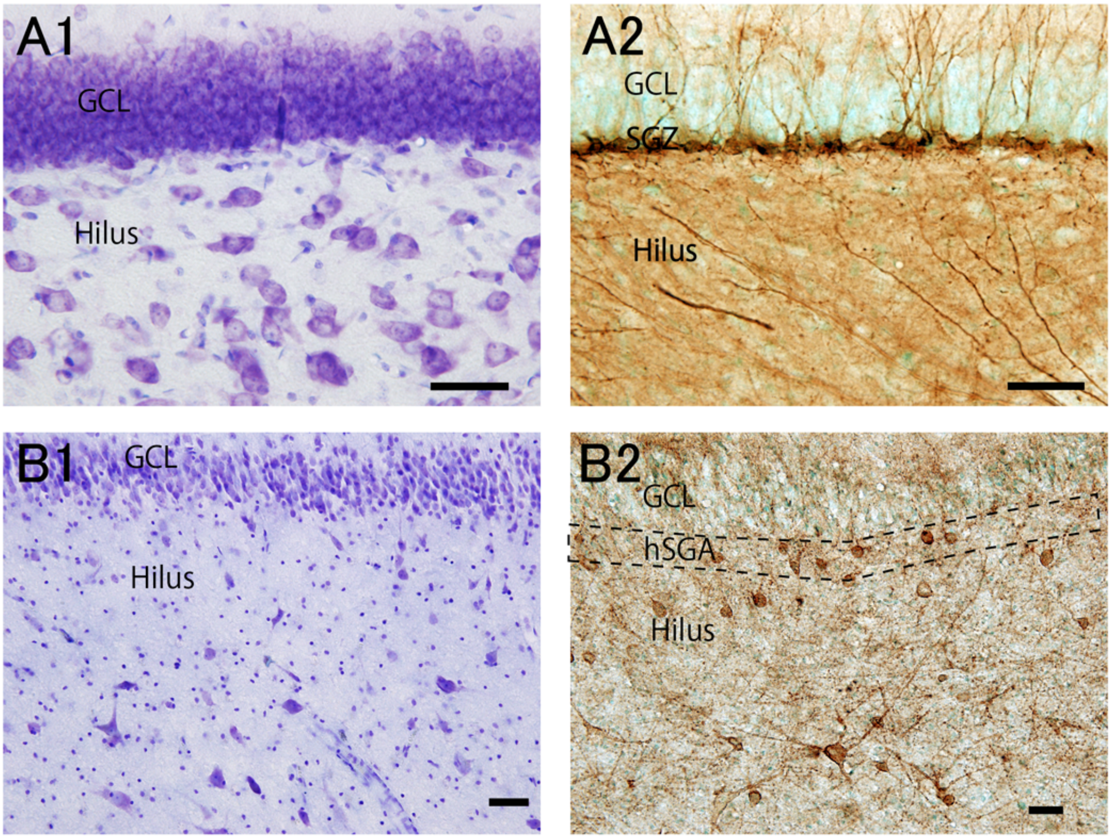

Supplemental Figure 2. Comparison between the rat subgranular zone (SGZ) (**A**) and the human subgranular area (hSGA) (**B**) using Nissl staining (**A1, B1**) and PSA-NCAM immunostaining (**A2, B2**). In rats, PSA-NCAM+ cells are confined to the narrow band below the granule cell layer (GCL), the so-called SGZ, whereas in humans, PSA-NCAM+ cells are distributed in a broad band below the GCL and also in the hilar region. For this reason, we refer to the putative human SGZ as the human subgranular area (hSGA), to avoid confusion with the narrow and distinct SGZ of rodents. In the present study, the hSGA was tentatively defined as a 50-µm wide region below the GCL (indicated by dashed lines), because the density of round or spindle-shaped cells resembling rodent neural progenitor cells was high compared with the lower area. Scale bars = 50 µm in A and B.

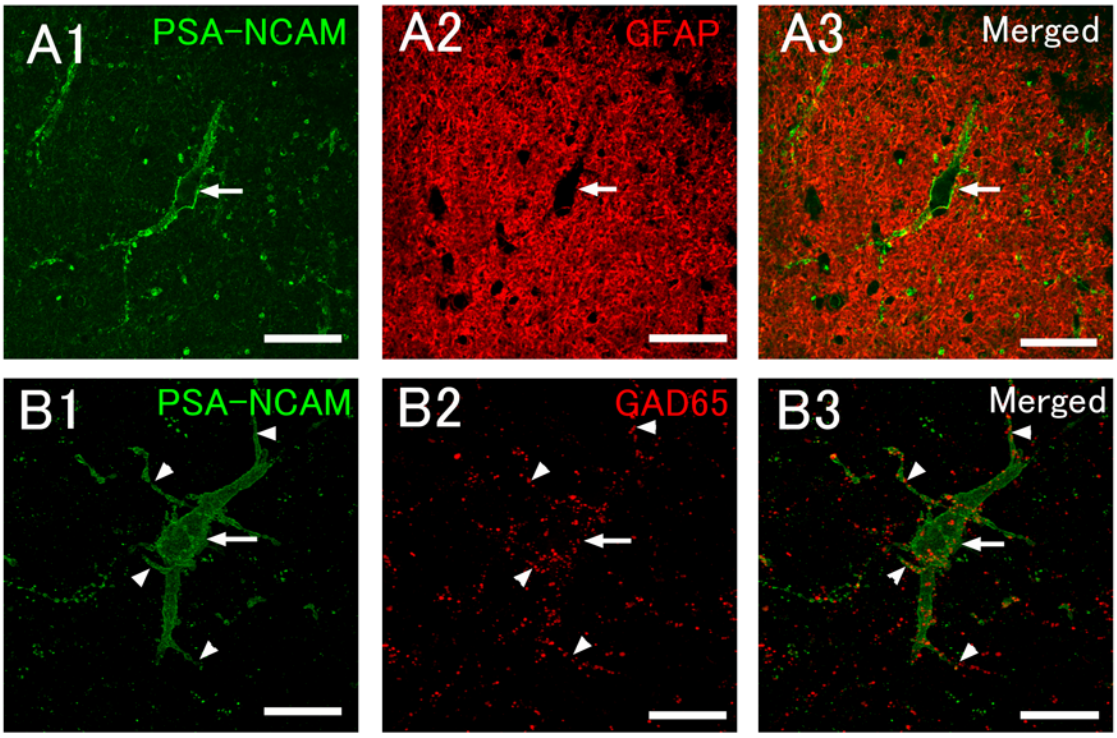

Supplemental Figure 3. (**A**) PSA-NCAM and GFAP immunohistochemistry in the hilus of an epileptic patient (EP9, see Supplemental Table 1). A PSA-NCAM+ aberrant cell (arrow) is negative for GFAP. (**B**) PSA-NCAM and GAD65 immunohistochemistry in the hilus of an epileptic patient (EP1). Z-stack images (**B1 – 3**) were reconstructed from 20 optical slices. GAD65+ terminals are distributed in the soma (arrow) and processes (arrowheads) of a PSA-NCAM+ aberrant cell, suggesting that PSA-NCAM+ aberrant cells are integrated into hippocampal neuronal circuits. Scale bars = 50 μm in A and B.

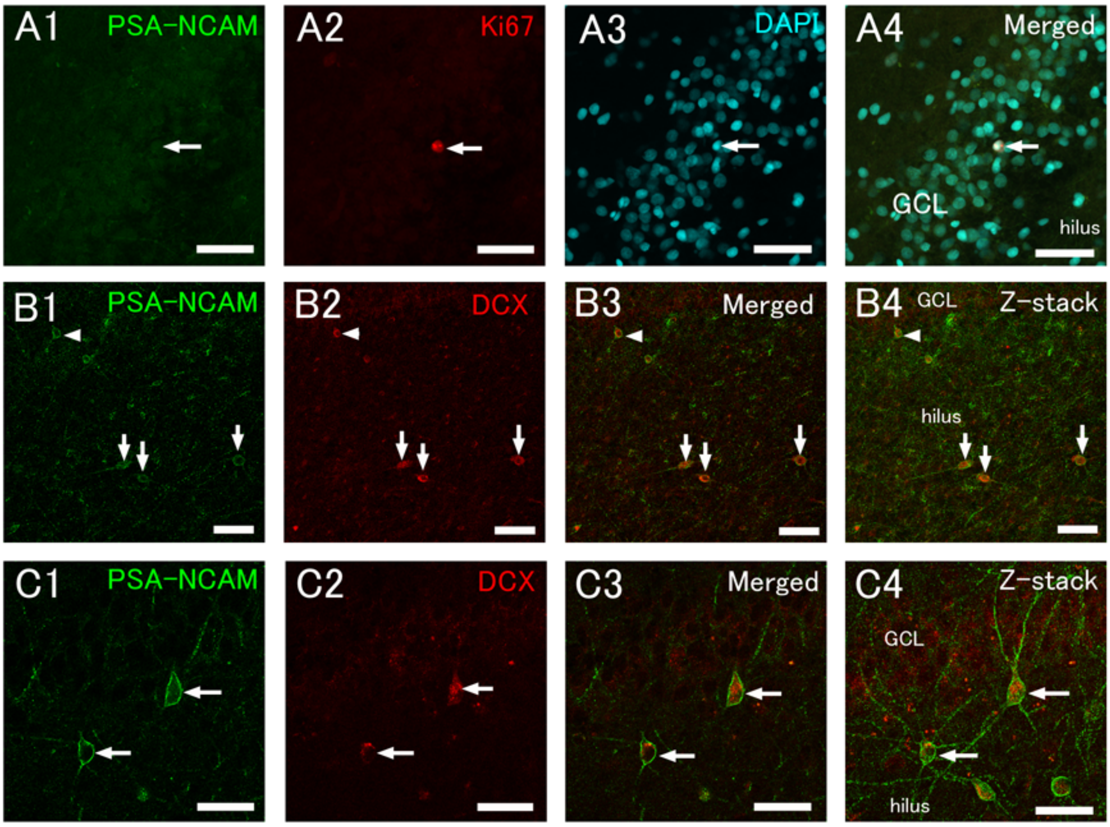

Supplemental Figure 4. Comparison of PSA-NCAM, DCX, and Ki67 immunostaining with or without citrate buffer pretreatment for antigen retrieval. Z-stack images of B4 and C4 were reconstructed from 10 and 21 optical slices, respectively. (**A**) Pretreatment with citrate buffer restored Ki67 immunoreactivity, but abolished PSA-NCAM immunoreactivity (arrows). (**B – C**) Comparison of the distribution of PSA-NCAM+ and DCX+ cells (arrows) without citrate buffer pretreatment. Images A and B were obtained from control patient CN2, and image C was from control patient CN3 (see Supplemental Table 1). Scale bars = 100 μm in B; 50 μm in A and C

Supplementary table 1.

Case No.	Gender	age at surgery	Onset	side of resection	HS types*	Clinical History and Others
Control						
CN1	M	16		left		glioma (fibrillary astrocytoma)
CN2	M	30		right		glioma
CN3	M	33		left		glioma
CN4	M	44		left		glioblastoma
CN5	M	49		right		cavernous angioma
CN6	M	49		right		astrocytoma
Patients with temporal lobe epilepsy						
mild granule cell dispersion						
EP1	F	9		right		Rusmussen encephalitis
EP2	M	17		right		
EP3	F	29		right	Type 2	
EP4	M	34		left		
EP5	M	37	22	right		
EP6	M	43	16	right		
sever granule cell dispersion						
EP7	F	19	7	left	Type 1	
EP8	M	35	10 mo	left	Type 3	
EP9	M	36	8	left	Type 1	
EP10	M	40	32	left	Type 1	
EP11	M	42	4-5	right	Type 1	used only in PSA-NCAM analysis
EP12	F	21	9	left	N.D.	used only in Ki67 analysis

Age of patients at surgery and onset of seizures is given in months (mo) or years. *The International League Against Epilepsy classifies hippocampal sclerosis (HS) into three types. Type 1 refers to severe neuronal cell loss and gliosis predominantly in the CA1 and CA4 regions, compared with CA1-predominant neuronal cell loss and gliosis (HS ILAE type 2), or CA4-predominant neuronal cell loss and gliosis (HS ILAE type 3)[1]. Control patient CN3 had a single seizure. N.D.: not determined

1. Blumcke I, Thom M, Aronica E, et al. International consensus classification of hippocampal sclerosis in temporal lobe epilepsy: A Task Force report from the ILAE Commission on Diagnostic Methods. *Epilepsia* 54:1315-1329 (2013).

Supplementary table 2. List of antibodies used in this study

	Species/Class	Dilution	Source	Catalog number
Primary antibody				
DCX	Guinea pig IgG	1 : 400	Chemicon (Merck,Darmstadt, Germany)	AB5910
DCX (C-18)	Goat IgG	1 : 400	Santa Cruz (Dallas, TX, USA)	sc-8066
GAD65	Mouse IgG	1 : 200	Sigma (Merck, Darmstatdt, Germany)	G1166
GFAP	Mouse IgG	1 : 200	Sigma (Merck, Darmstatdt, Germany)	G3893
HuB (HEL-N1)	Rabbit IgG	1 : 400	Sigma (Merck, Darmstatdt, Germany)	H-1538
Ki67 (MIB-1)	Mouse IgG	1 : 500	DAKO (Santa Clara, CA, USA)	M7240
PSA-NCAM (12E3)	Mouse IgM	1 : 1,000	Seki and Arai, 1991[52]	
Secondary antibody				
Goat IgG + Cy2	Donkey	1 : 200	Jackson ImmunoResearch (West Grove, PA, USA)	705-225-147
Guinea pig IgG + Cy3	Donkey	1 : 200	Jackson ImmunoResearch (West Grove, PA, USA)	706-166-148
Mouse IgG + Cy3	Donkey	1 : 200	Jackson ImmunoResearch (West Grove, PA, USA)	715-165-151
Mouse IgG + Cy5	Donkey	1 : 200	Jackson ImmunoResearch (West Grove, PA, USA)	715-175-151
Mouse IgM (μ) + Cy2	Goat	1 : 200	Jackson ImmunoResearch (West Grove, PA, USA)	115-225-075
Mouse IgM (μ) + biotin	Goat	1 : 200	Vectastain (Burlingame, CA, USA)	BA-2020
